# MicroRNA-665 mediates propofol-induced cell apoptosis in human stem cell-derived neurons

**DOI:** 10.1080/21655979.2019.1682105

**Published:** 2019-10-26

**Authors:** Lili Jiang, Fengyun Yang, Qin Zhao, Lixiao Pan

**Affiliations:** Department of Anesthesia, the Affiliated Hospital of Qingdao University, Qingdao, Shandong, China

**Keywords:** Propofol, neurotoxicity, apoptosis, miR-665

## Abstract

We aimed to evaluate the neurotoxicity and mechanisms of anesthetics propofol in hESC-derived neurons. Cell apoptosis in hESC-derived neurons' exposure to 4, 10 and 20 μg/mL propofol for 6 h was assessed using terminal deoxynucleotidyl transferase-mediated deoxyuridine triphosphate in situ nick end labeling (TUNEL) staining and microRNA-665 (miR-665) expression was assessed using quantitative reverse transcription polymerase chain reaction (qRT-PCR). miR-665 was overexpressed and knocked down using a miR-665 mimic and anti-665 transfection, respectively. The results showed that hESCs exposed to propofol showed a dose-dependent cell apoptosis, followed by the upregulation of miR-665 expression. Overexpression of miR-665 increased propofol-induced apoptosis in hESC cells. And targeting miR-665 decreased propofol-induced cell apoptosis in hESC cells. These data suggest that propofol induces cell death in hESC-derived neurons and the propofol-induced cell apoptosis may occur via miR-665-dependent mechanism.

## Graphical Abstract

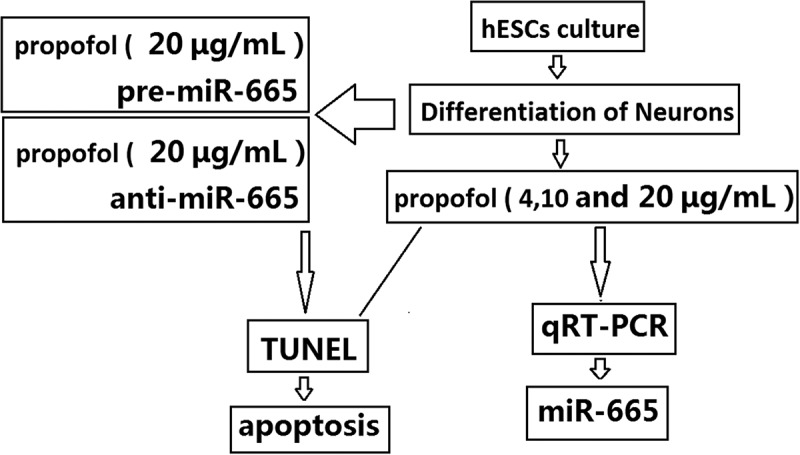

## Introduction

Propofol (2, 6-diisopropylphenol), known as ‘milk of anesthesia,’ is one of the most popular intravenous anesthetic agents in modern medicine. It is commonly used for the induction and maintenance of anesthesia and procedural and critical care sedation in children [1]. Propofol has been shown to induce neuronal cell death in neonatal rat and primate models [2,3]. Moreover, anesthetic exposure has been linked to learning disabilities and impaired cognitive function which has raised safety concerns regarding the use of anesthetics in children [4,5]. Therefore, it is critical to understand the effects of anesthetics on developing human neurons and its mechanisms of action in order to minimize any neurotoxic effects of propofol.

The mechanisms involved in developmental anesthetic-induced neurotoxicity are not well understood. Much of the research in the neurodegenerative field was performed in animal models with no direct evidence available in a human model. Additionally, for ethical reasons, it is not feasible to perform these studies on young children and the only human data available comes from a limited number of epidemiological studies [6].

Human embryonic stem cells (hESC) are pluripotent cell lines derived from the inner cell mass of early embryos. The ability to grow theoretically unlimited numbers of these stem cells and generate normal (i.e., nontransformed) cells of the human body makes them an exceptional tool for biomedical research and candidates for cell therapy of disease. In addition, the benefit of using hESCs lies in their ability to differentiate into any cell type making them a potentially powerful model of human physiology and pathophysiology. Therefore, neurons derived from hESCs are a valuable model to directly study the effects of anesthetics on immature, human-derived neurons.

Recent studies have demonstrated that microRNAs, as endogenous regulators of gene expression, were involved in the regulation of several biological processes, including cell adhesion, invasion, cell proliferation and apoptosis [7,8]. MicroRNAs have been implicated to play important roles in neurotoxicity conferred by ethanol, cocaine [9,10]. Huntington’s disease and brain injuries have all been linked to microRNA dysregulation [10,11]. In addition, brain injuries have also been linked to microRNA upregulation [12]. Despite rapid progress in understanding their functions, the precise roles of many miRNAs in the proliferation, migration, and differentiation of hESCs and hESC-derived hNPCs remain unknown.

Several miRNAs have been implicated in neuronal development in model organisms. Delaloy et al. have demonstrated a novel essential role for miR-9 in coordinating the proliferation and migration of hESCs-derived hNPCs at their early stage of maturation [13]. Li et al. have reported that propofol induces cell apoptosis in hESC-derived neurons via activation of the miR-206/PUMA signal pathway [14]. And Jiang et al. have reported that propofol inhibits hESCs neurogenesis through a mechanism involving the miR-141-3p/IGF2BP2 axis [15].

One microRNA, miR-665, has been shown to promoted cell apoptosis and can protect cardiac and intestinal function from ischemia/reperfusion injury [16,17]. Exposure of rodent developing hippocampal astrocytes to propofol was shown to induce miR-665 and promote cell apoptosis [18]. After screening microRNAs and finding that miR-665 was upregulated following exposure to propofol. In the present study, we hypothesized that the miR-665 plays a role in the increased cell apoptosis observed in the hESC-derived neurons following propofol.

## Materials and methods

### hESC culture

Mitotically inactivated mouse embryonic fibroblasts (MEFs) by mitomycin C (Sigma, Shanghai, China) were used as feeder cells to support the growth and maintenance of hESCs (H1 cell line, WiCell Research Institute Inc.). Inactivated MEFs were plated in 0.1% gelatin-coated 60 mm culture Petri dishes containing Dulbecco’s modified Eagle’s medium (DMEM) supplemented with 10% fetal bovine serum (Gibco) in a humidified incubator under normoxic condition (20% O_2_/5% CO_2_) at 37°C. The following day, hESCs were plated on the layer of MEFs with hESC culture medium and incubated in a hypoxic incubator (4% O_2_/5% CO_2_). hESC culture medium consisted of DMEM/F12 supplemented with 20% knock-out serum (Gibco, Guangzhou, China), 1% non-essential amino acids, 1% penicillin-streptomycin, 1 mM L-glutamine (Chemicon), 0.1 mM β-mercaptoethanol (Sigma), and 4 ng/mL human recombinant basic fibroblast growth factor (bFGF; Invitrogen, Guangzhou, China). The medium was changed daily. hESCs were passaged every 5–7 days using a mechanical microdissection method. hESCs with passage numbers between 70 and 80 were used in this study.

### Differentiation of neurons from hESCs

A 4-step directed differentiation protocol was used to generate neurons from the hESCs as previously described by our laboratory. Briefly, the hESCs were dissociated from the MEF cultures and the pellet of hESCs was resuspended in hESC media without bFGF and the cells were cultured in a normoxic incubator (20% O2/5% CO2, 37°C) on ultra-low attachment six-well plates (Corning Inc., Corning, NY). Embryoid bodies (EBs) were present in the cultures 1 day after digestion and the media was changed daily. Five days after dispase digestion, the cultures were switched to neural induction media consisting of DMEM/F12 supplemented with 1% N2 (Life Technologies), 1% nonessential amino acids, 1 mg/mL heparin (Sigma) and 5 ng/mL bFGF and the media was changed daily for an additional 4 days. The EBs were then plated down to 35 mm, matrigel-coated dishes. The media was changed every other day until rosette-like structures were present in the cultures (within 5 days of plating). The rosettes were manually selected using a pipette tip and transferred to new, matrigel-coated 35 mm dishes and cultured in neural expansion media containing DMEM/F12 supplemented with 2% B27 without vitamin A, 1% N2 (Life Technologies, Shanghai, China), 1% nonessential amino acids, 20 ng/mL bFGF and 1 mg/mL heparin. The neural stem cells (NSCs) present in the rosettes were passaged enzymatically every 5 days using Accutase (Innovative Cell Technologies, San Diego, CA). To generate neurons, NSCs were cultured in matrigel-coated dishes to about 95% confluency. Once confluent, the cultures were switched to neuron differentiation media. The media was changed every other day and neurons were used for these studies 2 weeks after initiation of differentiation media.

#### Propofol exposure

Brain concentrations of propofol in humans during anesthesia are believed to range between 4 and 20 μ g/mL. All experiments in this study were performed in 2-week-old hESC-derived neurons following a single exposure to 6 h of 4, 10 and 20 μ g/mL research grade propofol. The propofol was prepared as a 40 mg/mL stock solution in dimethyl sulfoxide (DMSO, Sigma-Aldrich, Hangzhou, China) and diluted to the working concentration in neurobasal media. An equal volume of DMSO was used as the vehicle control. The cells were cultured in matrigel-coated 60 mm culture dishes (500,000 cells/dish) or 12 mm coverslips (100,000 cells/coverslip) and all exposures were done in a humidified chamber at 37°C.

#### miR-665 knockdown or miR-665 overexpression

In order to manipulate the level of miR-665, 2-week-old hESC-derived neurons were transfected in six-well plates with 25 nM locked nucleic acid (LNA) anti-miR-665 to knockdown miR-665 or miR-665 overexpression by miR-665 mimic (pre-miR-665) transfection using lipofectamine (Invitrogen, Shanghai, China). The miR-665 mimic, inhibitor, and the respective negative controls were obtained from GenePharma (Shanghai, China). These concentrations were chosen based upon dose studies implicating the specified concentrations as sufficient to increase abundance or knockdown miR-665 in our cells. Twenty-four hours after transfection, a subset of cells was used to confirm miR-665 knockdown or miR-665 overexpression in the neurons by qRT-PCR. Following confirmation of knockdown of miR-665 or miR-665 or overexpression, the remaining cells were exposed to 20 μg/mL propofol for 6 h. The effect of miR-665 knockdown or miR-665 or overexpression on propofol-induced neurotoxicity in the hESC-derived neurons was analyzed by TUNEL staining.

### RNA extraction and real-time qRT-PCR

Total RNA was isolated from hESC-derived neurons with TRIzol reagent (Invitrogen, Shanghai, China) and reverse-transcribed into cDNA using oligo (dT)_15_ and ReverTra Ace reverse transcriptase (Toyobo, Osaka, Japan). NanoDrop 2000 (Thermo Scientific, Wilmington, DE, USA) was used to quantify amounts of mRNA. The qRT-PCR was performed using the ABI PRISM 7900 Fast Real-Time PCR system (Applied Biosystems, Shanghai, China) and the Power SYBR Green PCR Master Mix (Applied Biosystems, Shanghai, China) according to the manufacturer’s instructions. Mature miR-665 was detected by stem-loop qRT-PCR analysis using the Taqman Human MicroRNA Assay kits (Applied Biosystems, Shanghai, China). Small nucleolar RNU48 served as an endogenous reference RNA for normalizing the cellular content of other miRNAs.

Terminal deoxynucleotidyl transferase-mediated deoxyuridine triphosphate in situ nick end labeling (TUNEL)

hESC-derived neurons were fixed in 4% paraformaldehyde for 1 h at room temperature and then permeabilized in 0.2% Triton X-100/phosphate-buffered solution for 15 min. TUNEL staining was performed according to the manufacturer’s instructions (*In Situ* Cell Death Detection Kit, POD; Roche). Stained cells were analyzed using the BD FACSAria Cell Sorter.

#### Statistical analysis

The data were analyzed using SPSS 17.0 software (SPSS Inc., Chicago, IL, USA). All values are shown as means ± S.D. The Student’s *t*-test was used to determine the significance of differences in comparisons between two groups. The 0.05 level of confidence was considered statistically significant.

## Results

### Propofol induces cell death in hESC-derived neurons

**Figure 1.**
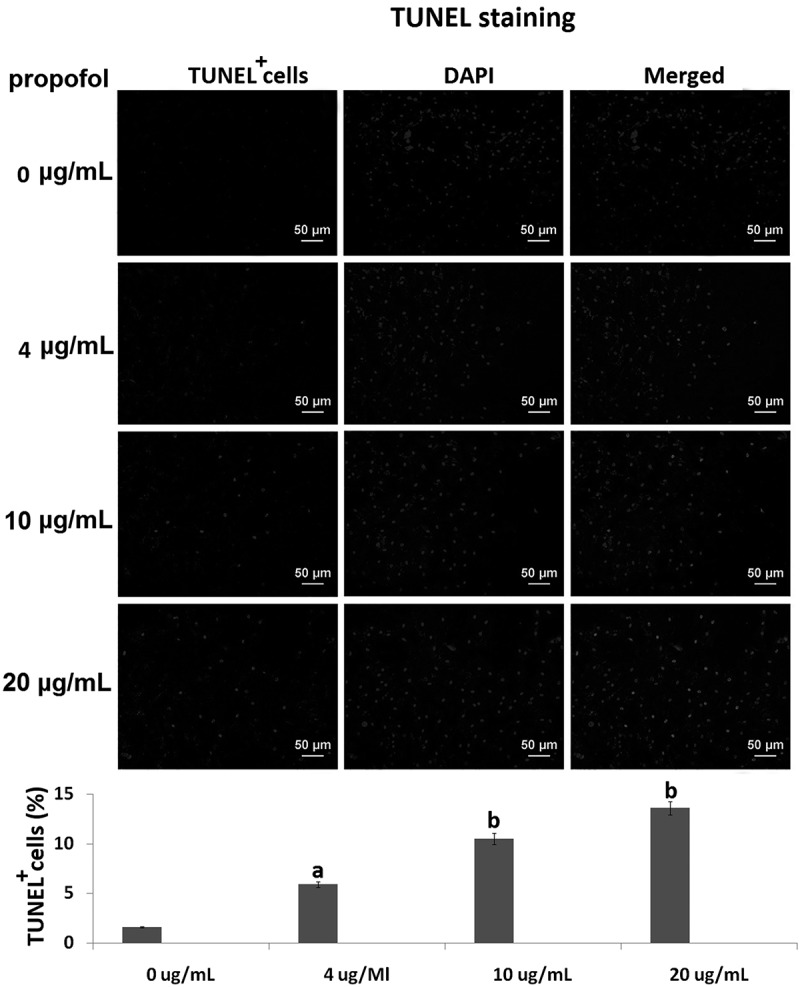
**The effects of propofol on apoptosis of hESC-derived neurons**. TUNEL assay revealed the percentages of TUNEL+ cells in the hESC-derived neurons treated with 4, 10 and 20 μg/mL propofol for 6 h. Scale bars, 50 mm. Statistically significant differences (^a^p < 0.05, ^b^p < 0.01).

TUNEL staining was used to assess cell death in hESC-derived neurons following propofol exposure. The cells were exposed 4, 10 and 20 μg/mL propofol for 6 h. The number of TUNEL-positive cells was (5.17 ± 0.87)%, (10.8 ± 2.3) % and (13.5±2.17) %, which was gradually increased when compared to the control cells (1.52 ± 0.35)% (Figure 1, vs control, ^a^p < 0.05,^b^p < 0.01).

### miR-665 is upregulated in hESC-derived neurons following propofol exposure

**Figure 2.**
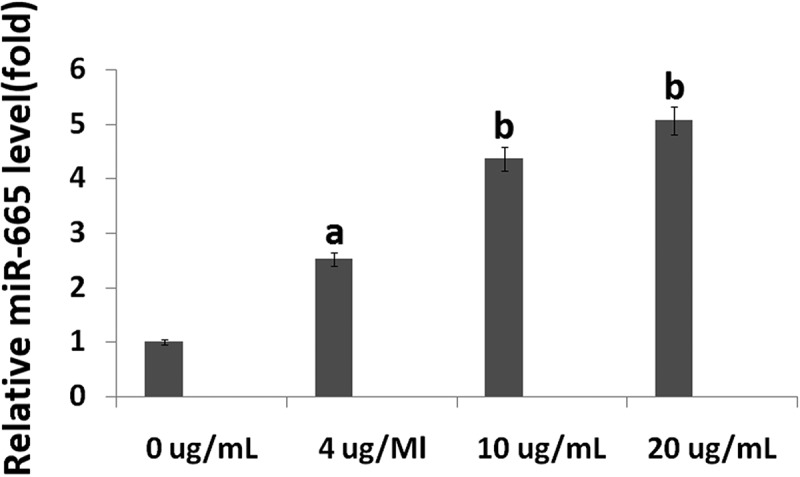
**The effects of propofol on miR-665 expression in hESC-derived neurons**. The qRT-PCR assay revealed the miR-665 expression levels in the hESC-derived neurons treated with 4, 10 and 20 μg/mL propofol for 6 h. Statistically significant differences (^a^p < 0.05, ^b^p < 0.01).

Samples were taken following 6 h of propofol exposure to determine miR-665 expression. The qRT-PCR assay showed that 4, 10 and 20 μg/mL propofol for 6 h significantly upregulated miR-665 expression (2.52 ± 0.83), (4.36 ± 1.02) and (5.07 ± 1.07) compared to control, respectively (^a^p < 0.01, ^b^p < 0.001) (Figure 2).

### miR-665 overexpression promotes propofol-induced cell apoptosis

**Figure 3.**
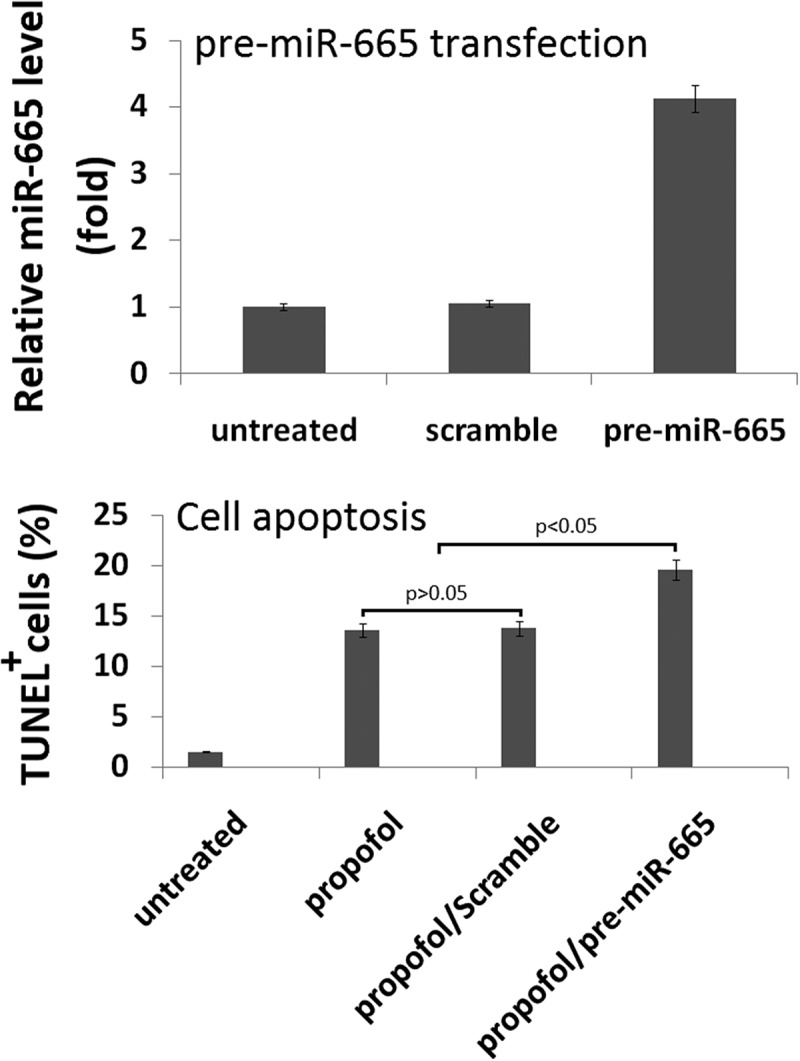
**Overexpression of miR-665 in hESC-derived neurons promotes the propofol-induced cell death**. Following 20 h of transfection with the miR-665 mimic, quantitative reverse transcription-PCR was used to confirm the overexpression of miR-665. miR-665 expression was significantly elevated following transfection with the miR-665 mimic when compared to scramble-treated cells. Overexpression of miR-665 significantly promotes the increase in TUNEL-positive cells following exposure to 6 h of 20 μg/mL propofol.

To evaluate the role of miR-665 in the propofol-induced neurotoxicity observed, a mimic was used to artificially overexpress miR-665 in the hESC-derived neurons. Following 20 h of transfection with the miR-665 mimic (pre-miR-665), the expression of miR-665 was confirmed using qRT-PCR. The levels of miR-665 were significantly higher in the cells transfected with miR-665 mimic when compared to scramble-treated cells (Figure 3).

To assess the effects of miR-665 overexpression on propofol-induced cell death, TUNEL staining was used. A single exposure to 6 h of 20 μg/mL propofol significantly increased the number of TUNEL-positive cells (13.6% ± 2.4%) when compared to untreated control cells (1.6% ± 0.3%). MiR-665 overexpression exacerbated the effects of the propofol and led to an increase in the number of TUNEL-positive cells (19.6% ± 1.4%), further confirming that miR-665 is playing a role in the propofol-induced toxicity observed in the hESC-derived neurons. Scramble transfection did not have an effect on the number of TUNEL-positive cells following propofol exposure (13.7% ± 2.4%) (Figure 3).

### Targeting miR-665 attenuates the propofol-induced cell death

**Figure 4.**
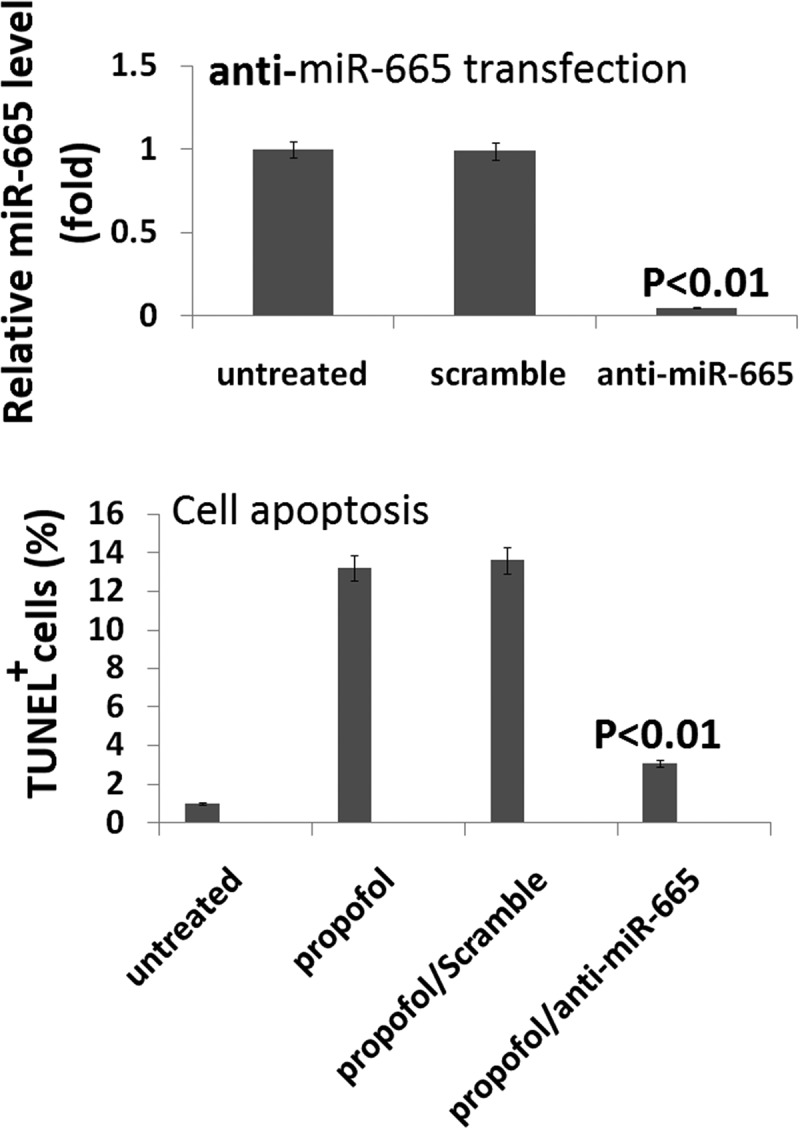
**Targeting miR-665 in hESC-derived neurons promotes the propofol-induced cell death**. Following 20 h of transfection with the anti-miR-665 mimic, quantitative reverse transcription-PCR was used to confirm the down-expression of miR-665. miR-665 expression was significantly decreased following transfection with the anti-miR-665 when compared to scramble-treated cells. Targeting miR-665 significantly inhibits the increase in TUNEL-positive cells following exposure to 6 h of 20 μg/mL propofol.

To further assess the role that miR-665 plays in propofol-induced neurotoxicity, a miR-665 antagomir (anti-miR-665) was used to knockdown miR-665 in the hESC-derived neurons using lipofectamine. The cells were transfected for 20 h with the anti-miR-665 or a scramble control and the knockdown was assessed by qRT-PCR. miR-665 expression was significantly reduced in the cells treated with the antagomir (0.05% ± 0.01%) when compared to the scramble-treated cells (100% ± 26.3%), confirming that the knockdown was successful (Figure 4).

To assess the effects of miR-665 knockdown on propofol-induced cell death, TUNEL staining was used. A single exposure to 6 h of 20 μg/mL propofol significantly increased the number of TUNEL-positive cells (13.4% ± 2.1%) when compared to vehicle-treated control cells (1.7% ± 0.3%). Knockdown of miR-665 reduced the effects of the propofol and led to a decrease in the number of TUNEL-positive cells (3.8% ± 0.9%), further confirming that miR-665 is playing a role in the propofol-induced toxicity observed in the hESC-derived neurons.

## Discussion

In our study, we examined the roles of propofol on human stem cell-derived neurons and the role of the miR-665 in the observed toxicity. We found that exposure to propofol induced significant cell apoptosis in the hESC-derived neurons and propofol upregulates the expression of miR-665 in the neurons. Downregulation of miR-665 significantly attenuated the increase in TUNEL-positive cells following propofol administration while miR-665 overexpression exacerbated the effects.

Studies have shown that anesthetics, when administered early in life, can lead to learning disabilities later in life in animal models [2,19–21]. In addition, studies performed in rodent and primate models have shown that propofol induces neuroapoptosis when administered for 5–6 h. Additionally, exposure of cultured neonatal rat hippocampal neurons to 5 h of 5 and 50 μM propofol was sufficient to induce cell death when compared to control-treated cells [18,22]. In 7-day-old rat pups administered six bolus injections, at 1 hour intervals of 20 mg/kg propofol; there was a significant increase in activated caspase-3 levels immediately following the exposure.

Twaroski et al. have reported that exposure of hESC-derived neurons to 6 h of 20 μg/mL propofol induced significant cell death and disorganization of the actin cytoskeleton [23]. In our study, we found that the 4, 10 and 20 μg/mL propofol for 6 h can induce significant cell apoptosis in 2-week-old hESC-derived neurons. Our findings are consistent with Li and Twaroski’s findings [18,23,24]. The mechanisms by which propofol induce neurotoxicity are not well understood. Sun et al. have reported that miR-665 is involved in the neurotoxicity induced by propofol via a caspase-3 mediated mechanism by negatively regulating BCL2L1 [25,26], suggesting miR-665 was the pro-apoptotic gene. Twaroski et al. have reported the propofol-induced cell death may occur via a STAT3/miR-21/Sprouty2-dependent mechanism [24]. However, the anesthetic agent sevoflurane induced hippocampal neuroapoptosis by miR-665 downregulation, suggesting miR-665 was the anti-apoptotic gene [27].

In our study, we found that the expression of miR-665 was significantly upregulated by propofol administration. miR-665 knockdown attenuates the propofol-induced cell death seen in the hESC-derived neurons, and overexpression of miR-665 exacerbated the toxic effects of propofol. These data indicate that miR-665 is playing an important role in the neuronal cell death.

### Conclusions

Our data suggest that propofol induces cell apoptosis in hESC-derived neurons by miR-665 upregulation. miR-665 overexpression exacerbates propofol-induced cell apoptosis and miR-665 silencing inhibits propofol-induced cell apoptosis in hESCs. Establishing the role of miR-665 in propofol-induced neurotoxicity could possibly pave the way for new research into possible neuroprotective strategies.

## Highlights

- hESCs represent a promising *in vitro* human model for studying anesthetic-induced neurotoxicity.
- Propofol-induced cell apoptosis in hESCs by miR-665 upregulation.
- miR-665 overexpression promotes propofol-induced cell apoptosis in hESCs.
- miR-665 silencing inhibits propofol-induced cell apoptosis in hESCs.
